# Memories Ruined

**Published:** 1879-06

**Authors:** 


### MEMORIES RUINED.

The general tendency of the manner in which the public schools are conducted in New-York city is to make study a mere mechanical action of the memory and then destroy it.

The ambition of the child is to recite his lesson correctly and by whatever means, fair or foul, he can escape the terrible sentence of "failure" he will adopt; he will cheat, prevaricate, and lie remorselessly; they do it every day; nine out of ten of all of them will do it; and that the terror the sentence of "failure" should drive to these things is a moral enormity which ought to be abated, in our opinion, even if every school house was burned down, and every trustee in it.

So great is the desire to "say the lesson" acceptably that the whole effort and being of the child is expended in the direction of impressing the lesson on the memory and after the lesson is said there is a reaction on the mind and body of the child, which makes a single thought about the past lesson a burden, and it is instinctively shunned and the result is in very many cases, that the next day the lesson is in a measure forgotten. A single illustration in our own hearing is sufficient; four children had been going to the public school; the younger, five years, in coming to a lesson it seemed a little hard, and each of the other three children of greater age were applied to, and every one answered "I have forgotten it." Whereas if the principle had ever been impressed on the mind, it could not have been forgotten; similar exhibitions as to the fact of past studies having escaped the memory have been noticed in a great multitude of cases.

The tyranny which the dread of bad marks and failures exercises on the mind of the children of the public schools is not merely severe; it is terrible; but we see no hope of a change until the parents become wise enough to care at least as much for the wellfare of their children at school as for their canary birds, horses, cats and poodle dogs.

